# The complete chloroplast genome of *Camellia vietnamensis*, an economic shrub producing edible seed oil

**DOI:** 10.1080/23802359.2019.1681305

**Published:** 2019-10-24

**Authors:** Tao Lyu, Yupeng Wang, Zhikang Hu, Xinlei Li, Jiyuan Li, Zhengqi Fan, Ning Ye, Hengfu Yin

**Affiliations:** a State Key Laboratory of Tree Genetics and Breeding, Research Institute of Subtropical, Forestry, Chinese Academy of Forestry, Hangzhou, China;; b Key Laboratory of Forest Genetics and Breeding, Research Institute of Subtropical, Forestry, Chinese Academy of Forestry, Hangzhou, China;; c College of Information Science and Technology, Nanjing Forestry University, Nanjing, China

**Keywords:** *Camellia*, chloroplast, genome sequencing, edible oil, phylogeny

## Abstract

*Camellia vietnamensis* is an economic woody plant producing high-value edible oils, which is commonly found and cultivated in south areas of China. To provide genetic information for future genetic research, we have sequenced and assembled the complete chloroplast (cp) genome of *C. vietnamensis* based on the Illumina Hiseq platform. The total genome size is 161,958 bp in length with 37% GC, which contains a large single copy (LSC, 86,657 bp) region, a small single copy (SSC, 13,347 bp) region, and a pair of inverted repeat (IRs, 30,977 bp) regions. It is comprised of 81 protein-coding genes, 44 transfer RNAs and 4 ribosomal RNAs. To obtain the phylogeny relationship, the cp genome of *C. vietnamensis* has been compared with other *Camellia* species; the results indicate that *C. vietnamensis* is closely related to *C. taliensis*. This study provides fundamental information of *C. vietnamensis* cp genome, and it is valuable to the molecular phylogenetic and genetic diversity analyses in future.

*Camellia vietnamensis* is an economic woody plant, found mainly in Liuzhou city, Guangxi Province, China; it has now been widely used in producing edible oil from the seed kernels (Liu et al. 2018). However, the genomic information of this plant species remains poorly understood. The chloroplast (cp) genome has been extensively used to obtain the knowledge of phylogeny and genetic diversity, especially in species with limited genomic resources, due to the high conservation of sequences, structure and compositions (Wicke et al. 2011). Here, we performed the high-throughput sequencing and described the assembly and annotation details of the *C. vietnamensis* cp genome (NCBI Accession Number: MN172193). The voucher specimen (CV_01) was reserved in State Key Laboratory of Tree Genetics and Breeding, Research Institute of Subtropical Forestry, Chinese Academy of Forestry.

The samples of *C. vietnamensis* were collected from Liuzhou city (Guangxi, China; Coordinates: 109°42′E, 24°33′N; Altitude: 91 m). Total genomic DNA was extracted from fresh leaf tissue samples using MiniBEST plant Genomic DNA Extraction Kit (Takara, Dalian, China). And the DNA concentration quality was controlled higher than 20 ng/µL (total mass was higher than 100 ng) by NanoDrop 2000 (Thermo Fisher Scientific, USA). To perform sequencing, the Illumina libraries were constructed using TruSeq DNA sample Preparation kit (Illumina, San Diego, CA, USA), and 2 × 150 pair-end sequencing was carried out on the Illumina Hiseq 2500 platform (Illumina, San Diego, CA, USA) at Genesky Biotechnologies (Shanghai, China).

We originally gained 25,960,736 reads and 25,960,736 bases. And we retrieved 2,432,5026 sequences and 3,504,844,406 bases after quality control using Trimmomatic (Bolger et al. 2014). The cp genome of *Camellia japonica* (NCBI accession Number: NC_036830.1) was selected as the reference genome sequence to filter the reads by Bowtie2 (Langmead and Salzberg 2012). Samtools software was used to calculate the sequencing depth (Li et al. 2009). Ultimately, 461,512 plastid reads were matched with an average coverage of 23 folds. Fast-Plast v1.2.8 was used to assemble the cp genome of *C. vietnamensis* (https://github.com/mrmckain/Fast-Plast) (McKain and Wilson 2018). The annotation of the genome was performed using the programme OrganellarGenomeDRAW (Lohse et al. 2013), and then manually confirmed through comparing with the cp genome of *C. japonica*.

The assembled complete cp genome of *C. vietnamensis* was 161,958 bp in length with 37% GC. It displayed the typical quadripartite structure, containing a large single-copy (LSC) region of 86,657 bp, a small single-copy (SSC) region of 13,347 bp, and a pair of inverted repeats (IRs) of 30,977 bp. In total it contained 129 functional genes, comprised of 81 protein-coding genes, 44 transfer RNAs and 4 ribosomal RNAs.

We performed a phylogenetic analysis using seventeen complete cp genomes of *Camellia* species. The conserved protein sequences were extracted for alignment (Wang et al. 2018); MEGA v7.0.14 was used to determine the phylogenetic relationships by the Neighbor-joining method (Kumar et al. 2016). It was found that *C. vietnamensis* was closely related to *Camellia taliensis* (Figure 1). The complete cp genome of *C. vietnamensis* would be utilised in molecular phylogenetic and genetic diversity analysis of camellias in the future.

**Figure 1.**
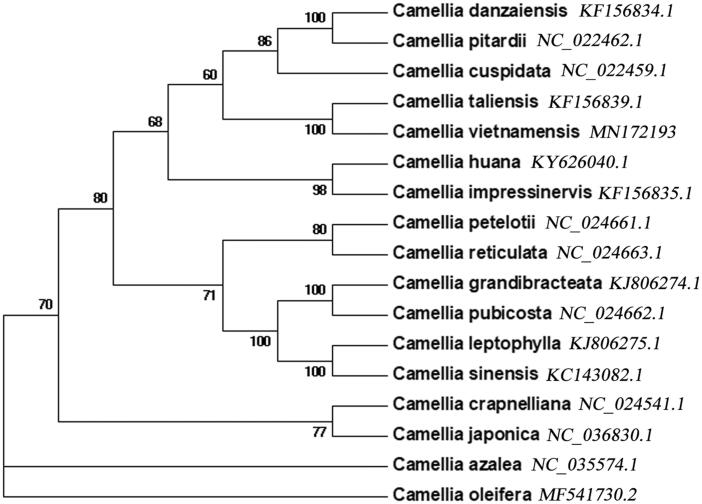
The Neighbor-joining phylogenetic tree for *C. vietnamensis* with other *Camellia* species based on conserved protein sequences of cp genomes.
